# Decreased free D-aspartate levels in the blood serum of patients with schizophrenia

**DOI:** 10.3389/fpsyt.2024.1408175

**Published:** 2024-07-10

**Authors:** Martina Garofalo, Giuseppe De Simone, Zoraide Motta, Tommaso Nuzzo, Elisa De Grandis, Claudio Bruno, Silvia Boeri, Maria Pia Riccio, Lucio Pastore, Carmela Bravaccio, Felice Iasevoli, Francesco Salvatore, Loredano Pollegioni, Francesco Errico, Andrea de Bartolomeis, Alessandro Usiello

**Affiliations:** ^1^ CEINGE Biotecnologie Avanzate “Franco Salvatore”, Naples, Italy; ^2^ Department of Environmental, Biological and Pharmaceutical Sciences and Technologies, Università degli Studi della Campania “Luigi Vanvitelli”, Caserta, Italy; ^3^ Section of Psychiatry, Laboratory of Translational and Molecular Psychiatry and Unit of Treatment-Resistant Psychosis, Department of Neuroscience, Reproductive Sciences and Odontostomatology, University Medical School of Naples “Federico II”, Naples, Italy; ^4^ ”The Protein Factory 2.0”, Dipartimento di Biotecnologie e Scienze della Vita, Università degli Studi dell’Insubria, Varese, Italy; ^5^ Department of Neuroscience, Rehabilitation, Ophthalmology, Genetics, Maternal, and Child Health - DINOGMI, University of Genoa, Genoa, Italy; ^6^ Center of Translational and Experimental Myology, Istituto di Ricovero e Cura a Carattere Scientifico (IRCCS) Istituto Giannina Gaslini, Genoa, Italy; ^7^ Department of Maternal and Child Health, Unità Operativa semplice di Dipartimento (UOSD) of Child and Adolescent Psychiatry, Azienda Ospedaliera Universitaria (AOU) Federico II, Naples, Italy; ^8^ Dipartimento di Medicina Molecolare e Biotecnologie Mediche, Università degli Studi di Napoli “Federico II”, Naples, Italy; ^9^ Department of Medical and Translational Sciences, Child Neuropsychiatry, Federico II University, Napoli, Italy; ^10^ Centro Interuniversitario per Malattie Multigeniche e Multifattoriali e loro Modelli Animali (Federico II, Naples; Tor Vergata, Rome and “G. D’Annunzio”, Chieti-Pescara), Naples, Italy; ^11^ Dipartimento di Agraria, Università degli Studi di Napoli “Federico II”, Portici, Italy

**Keywords:** D-serine, D-aspartate, treatment-resistant, antipsychotics, schizophrenia, autism spectrum disorder

## Abstract

**Introduction:**

Schizophrenia (SCZ) and autism spectrum disorder (ASD) are neurodevelopmental diseases characterized by different psychopathological manifestations and divergent clinical trajectories. Various alterations at glutamatergic synapses have been reported in both disorders, including abnormal NMDA and metabotropic receptor signaling.

**Methods:**

We conducted a bicentric study to assess the blood serum levels of NMDA receptors-related glutamatergic amino acids and their precursors, including L-glutamate, L-glutamine, D-aspartate, L-aspartate, L-asparagine, D-serine, L-serine and glycine, in ASD, SCZ patients and their respective control subjects. Specifically, the SCZ patients were subdivided into treatment-resistant and non-treatment-resistant SCZ patients, based on their responsivity to conventional antipsychotics.

**Results:**

D-serine and D-aspartate serum reductions were found in SCZ patients compared to controls. Conversely, no significant differences between cases and controls were found in amino acid concentrations in the two ASD cohorts analyzed.

**Discussion:**

This result further encourages future research to evaluate the predictive role of selected D-amino acids as peripheral markers for SCZ pathophysiology and diagnosis.

## 1 Introduction

Several lines of evidence suggest a neurodevelopmental origin of neuropsychiatric disorders such as schizophrenia (SCZ) (1, 2) and autistic spectrum disorder (ASD) (3), both considered polygenic and multifactorial in origin (4, 5). Compelling evidence from neurochemical and genetic investigations points to abnormal glutamate synaptic features as a major underpinning of SCZ and ASD pathophysiology (6–10). Accordingly, SCZ and ASD show pronounced synapse alterations (11, 12) and aberrant cortical-subcortical brain connectivity (13, 14). Specifically, glutamatergic dysfunction has been reported both at the level of transporters (15) and receptors (16, 17) as well as at glutamatergic postsynaptic density (PSD) macromolecular protein assembly, also coherent with the evidence of abnormal Shank, Homer, and PSD95 expression (12, 18–21).

The relevance of glutamatergic system dysfunction in SCZ pathophysiology has been further expanded by the discovery of altered metabolism of two free amino acids in the atypical D-configuration, D-serine (D-Ser) and D-aspartate (D-Asp), in SCZ (22–26). Both D-amino acids modulate ionotropic NMDA receptor (NMDAR)-dependent transmission by acting as endogenous co-agonist (D-Ser) and agonist (D-Asp) at the glycine site of GluN1 (D-Ser) and the glutamate site of GluN2 (D-Asp) subunits of NMDARs (27–29). Furthermore, free D-Asp stimulates metabotropic Glu5 receptors (mGluR5) coupled to polyphosphoinositide hydrolysis in neonate rat brain slices, thus suggesting a functional involvement for this D-amino acid also on mGluR5 signaling during early postnatal life (30). Dedicated enzymatic systems regulate the endogenous levels of D-Ser and D-Asp. D-Ser is synthesized from L-Ser by serine racemase (SR) (31) and degraded by D-amino acid oxidase (DAAO) (32). Conversely, the enzymatic machinery responsible for D-Asp biosynthesis has not yet been fully identified even though SR can produce to some extent D-Asp in the forebrain (33, 34), while its degradation is catalyzed by D-aspartate oxidase (DASPO or DDO) activity (32, 35). Besides endogenous biosynthesis, recent studies highlighted that both dietary intake and gut microbiota contribute to the endogenous pool of D-amino acids (36–38). Interestingly, D-Ser and D-Asp display distinct ontogenetic profiles in the mammalian brain. Cerebral D-Ser levels are constantly elevated during lifetime and decrease in the elderly stage, due to reduced SR expression (39). On the other hand, D-Asp levels are substantially high in the developing brain and drastically decrease in adulthood (27, 29, 40). In agreement with the hypothesis of NMDAR hypofunction in SCZ (41, 42), previous evidence indicates lower D-Ser levels in the serum and cerebrospinal fluid of patients with SCZ (43–56). Furthermore, genetic studies revealed an association between SCZ and *serine racemase* (*SR*), and *D-amino acid oxidase* (*DAAO*) genes (57–61), as well as *G72* gene, encoding the main DAAO modulator, pLG72 (62–64). Moreover, both D-Ser supplementation and DAAO inhibition have shown beneficial effects in modulating mismatch negativity response, cognition and extrapyramidal side-effects linked to antipsychotic treatment in SCZ patients (65–71). Also, preclinical studies revealed that reduced D-Ser levels in *Sr* knockout mice, a model of NMDAR hypofunction, show different phenotypes relevant to SCZ (72–74).

Despite the role of D-Asp in the mammalian central nervous system (CNS) has been so far much less detailed than that of D-Ser, preclinical and *post-mortem* findings in the last decade have suggested an involvement of this endogenous NMDAR agonist in SCZ pathophysiology (25, 26). In this regard, neurochemical analyses performed in two different *post-mortem* brain cohorts have shown that D-Asp content (detected in the order of tens of nmol/g tissue) decreases by about 30–40% in the prefrontal cortex (PFC) of SCZ patients, compared to non-psychiatric subjects (75, 76), accompanied by a concomitant reduction of D-Asp/total Asp ratio (76). Alteration of the latter parameter, representing an index of the metabolic conversion rate of L-Asp into its derivative, D-Asp, suggests the existence of a homeostatic cerebral dysregulation in the D-enantiomer metabolism, as also indicated by the increases in either enzymatic DDO activity (76) or *DDO* gene expression (75) in SCZ *post-mortem* PFC. Additionally, a recent study based on a machine learning hypothesis-free algorithm identified in the *post-mortem* dorsolateral PFC (DLPFC) a stable cluster of molecules of the glutamatergic synapse, including D-Asp/total Asp ratio and D-Ser, that discriminate SCZ patients from non-psychiatric controls (77). In line with the involvement of D-Asp metabolism deregulation as a potential vulnerability factor in the onset of neurodevelopmental disorders, we have recently identified a duplication of a chromosome 6 region, including the entire *DDO* gene, in a young patient with severe intellectual disability, thought disorders and behavioural abnormalities reminiscent of ASD and SCZ symptomatology (78). Consistent with this clinical evidence, we have also found that *Ddo* gene duplication and the consequent constitutive depletion of cerebral D-Asp levels in mice (79) produce abnormal corticogenesis, decrease cortico-striatal gray matter volume and induce social recognition memory deficit in adulthood (78).

Based on D-Ser and D-Asp involvement in NMDAR and mGluR5 signaling, in the present work, we analyzed the levels of these atypical molecules and other main neuroactive amino acids acting on glutamatergic neurotransmission in the serum of ASD and SCZ patients and their respective control groups.

## 2 Methods

### 2.1 Demographic and clinical characteristics of patients with schizophrenia

Blood serum samples were obtained from SCZ patients (*n* = 26) and non-psychiatric controls (*n* = 13). Patients with schizophrenia were recruited at the A.O.U. “Federico II” hospital of Naples over 6 months and diagnosed according to the Diagnostic and Statistical Manual of mental disorders, Fifth Edition (DSM-5) (80). Inclusion criteria for patients were: age 18–60 years; no evidence of worsening psychotic symptoms in the previous 6 months; absence of other major systemic, psychiatric (e.g., addictive disorders, frequent substance use in the 6 months prior to enrollment, etc.), or neurological disorders. Healthy controls were sex-matched individuals with no history of neurological, psychiatric, or systemic conditions or family psychiatric history. SCZ patients were divided into two groups according to treatment resistance: non-treatment-resistant schizophrenia (nTRS; *n* = 13) and treatment-resistant schizophrenia (TRS; *n* = 13). The treatment resistance condition was defined as a failure of at least two different antipsychotic regimens, each administered for > 6 weeks and at an optimal dose, according to the modified Treatment Response and Resistance in Psychosis Working Group Consensus criteria (81). All TRS patients were under treatment with clozapine while nTRS patients were treated with different conventional antipsychotics, such as olanzapine, risperidone, haloperidol, amisulpride, promazine, paliperidone and aripiprazole. Clinical data were collected within 1 month from the blood sample and included the severity of psychotic symptoms measured by the Positive and Negative Syndrome Scale (PANSS) (82) and cognitive performances assessed by the Brief Assessment of Cognition in Schizophrenia (BACS) (83). Demographic characteristics are reported in **Table 1**. Phlebotomy was conducted by a psychiatric nurse; collection was performed in fasting status, in the morning before breakfast. Serum was separated by centrifugation and stored at −80°C until analysis. Written informed consent was obtained from all subjects, according to the Declaration of Helsinki. The study was approved by the Ethics Committee of the University “Federico II” of Naples (protocol number: 195/19).

**Table 1:** Demographic characteristics of schizophrenia and control patients enrolled in the blood serum collection.

Demographic information	Control (*n*=13)	nTRS (*n*=13)	TRS (*n*=13)	Control *vs* nTRS *vs* TRS		
				*Statistic*	*p-value*	Effect size
Age (years)	28 [21; 40]	47 [22; 60]	34 [25; 57]	F(2, 36) = 10.688	0.0002^a^	η²p = 0.37
Sex (male/female)	6/7	10/3	11/2	*Χ*² (2, *N* = 39) = 5.0556	0.0798^b^	Cohen’s w = 0.36

Values are expressed as median [minimum; maximum] for age. For sex number of subjects (n) is indicated. Statistical analyses were performed by ^a^one way ANOVA or ^b^Chi-square test. nTRS, non-treatment-resistant schizophrenia; TRS, treatment-resistant schizophrenia.

### 2.2 Demographic and clinical characteristics of patients with autism spectrum disorder

Blood serum samples were obtained from two different Italian hospitals: A.O.U. “Federico II”, Naples, Italy (ASD, *n* = 33; Control, *n* = 6) and Istituto Giannina Gaslini, Genoa, Italy (ASD, *n* = 20; Control, *n* = 24). Participants from A.O.U. “Federico II” were consecutive samples of children and adolescents, along 6 months, referred to the Department of Pediatrics — Unit of Child and Adolescent Neuropsychiatry, for an evaluation in a clinical hypothesis or revaluation of ASD. All the subjects received a full assessment, including a complete history (pregnancy, childbirth, psychomotor development), structured clinical interviews and validated observations [Autism Diagnostic Observation Schedule-2 (84), Griffiths Mental Development Scale (85) or Leiter International Performance Test-Revised (86), Vineland Adaptive Behavior Scales—II edition (87)]. Diagnosis of ASD was formulated according to DSM-5 (80).

About 60 subjects were evaluated; study participants included 33/60 ASD subjects, whose parents signed an informed consent form to participate in the study. Subjects were aged between 18 and 189 months, both males (*n* = 28) and females (*n* = 5). Inclusion criteria were a clinical diagnosis of ASD, less than 18 years of age; exclusion criteria included: epilepsy diagnosis or other neurological disorders; psychiatric comorbidity (e.g. obsessive-compulsive disorder, psychosis, etc.), other chronic diseases (e.g. chronic intestinal diseases, malabsorption, etc.).

Six healthy typically developed subjects were recruited as a control group; inclusion criteria were the absence of psychiatric diagnosis, less than 18 years of age. For the control group, the same exclusion criteria were used.

The enrolled subjects followed routine clinical procedures for outpatients, from which data were collected. Each patient was also investigated by blood samples, as per routine procedures during clinical evaluation. Blood samples were collected in the hospital for both ASD and control children. Phlebotomy was conducted by a pediatric nurse, collection was made in fasting status, in the morning before breakfast. Serum was separated by centrifugation and stored at −80°C until analysis. The study was conducted according to the principles of the Declaration of Helsinki; ethical approval was obtained by the Ethics Committee of the University Federico II of Naples (220/18). Written informed consent was collected from parents or legal guardians of enrolled children for both clinical information collection and data acquisition and treatment.

Participants from Istituto Giannina Gaslini were consecutive samples of children and adolescents, referred to the Child Neuropsychiatry Unit Day Hospital for a third-level neuroradiological, biochemical, metabolic and genetic evaluation in a clinical diagnosis of ASD. All the subjects received a full assessment, including a complete history (pregnancy, childbirth, psychomotor development), structured clinical interviews and validated observations [Autism Diagnostic Observation Schedule-2 (84), Autism Diagnostic Interview (Lord et al., 1994), Griffiths Mental Development Scale (85), Wechsler Intelligence Scale for Children – fourth edition (Wechsler D., 2003) or the Wechsler Preschool and Primary Scale of Intelligence - III edition - WPPSI-III (Wechsler D., 2002), Vineland Adaptive Behavior Scales—II edition (87)]. Diagnosis of ASD was formulated according to DSM-5 (80).

Study participants included 20 ASD subjects aged between 3 years and 6 months and 11 years and 4 months, 19 males and 1 female. Inclusion criteria were a clinical diagnosis of ASD, less than 18 years of age; while exclusion criteria were the presence of other psychiatric diagnosis, epilepsy or other chronic diseases. Twenty-four developing normal children were recruited as a control group; inclusion criteria were the absence of psychiatric diagnosis, less than 18 years of age. For the control group, the same exclusion criteria were used.

Blood collection was made in fasting status, in the morning. Serum was separated by centrifugation and stored at −80°C until analysis. The study was conducted according to the principles of the Declaration of Helsinki; ethical approval was obtained by the Ethics Committee of the Liguria Region (N. CET - Liguria: 437/2023 - DB id 13411). Demographic characteristics of ASD and control individuals from both hospitals’ cohorts, such as age and sex distribution, are reported in **Table 2**. The hospitals involved in the study were chosen for the presence of SCZ outpatient clinics including a referral center for treatment-resistant psychosis (adult patients at University Federico II - Psychiatry Section) and for being regional centers of child psychiatry referral (Child Neuropsychiatry at Istituto Giannina Gaslini of Genoa and A.O.U. Federico II Neuropsychiatry Section of Naples).

**Table 2:** Demographic characteristics of ASD and control patients enrolled by two different Italian hospitals.

Demographic information	Istituto Giannina Gaslini			A.O.U. “Federico II”		
	Control (*n*=24)	ASD (*n*=20)	*Statistic*	Control (*n*=6)	ASD (*n*=33)	*Statistic*
Age (years)	9.8 [3.1; 24.8]	6.1 [3.5; 11.3]	*t*(42) = -3.414; *p* = 0.001^a^; Cohen’s d = -1.03	13 [5; 17]	5.4 [0.4; 15.7]	*t*(36) = -3.075; *p* = 0.004^a^; Cohen’s d = -1.37
Sex (male/female)	24/0	19/1	*Χ²* (1, *N* = 44) = 0.009; *p* = 0.926^b^; Cohen’s w = 0.17	1/5	28/5	*Χ²* (1, *N* = 39) *=* 9.060*; p*=0.003^b^; Cohen’s w = 0.56

Values are expressed as median [minimum; maximum] for age. For sex number of subjects (n) is indicated. Statistical analyses were performed by ^a^ Student’s t test or ^b^ Chi-square test. ASD, Autism spectrum disorder.

### 2.3 HPLC analysis

**Figure 1:**
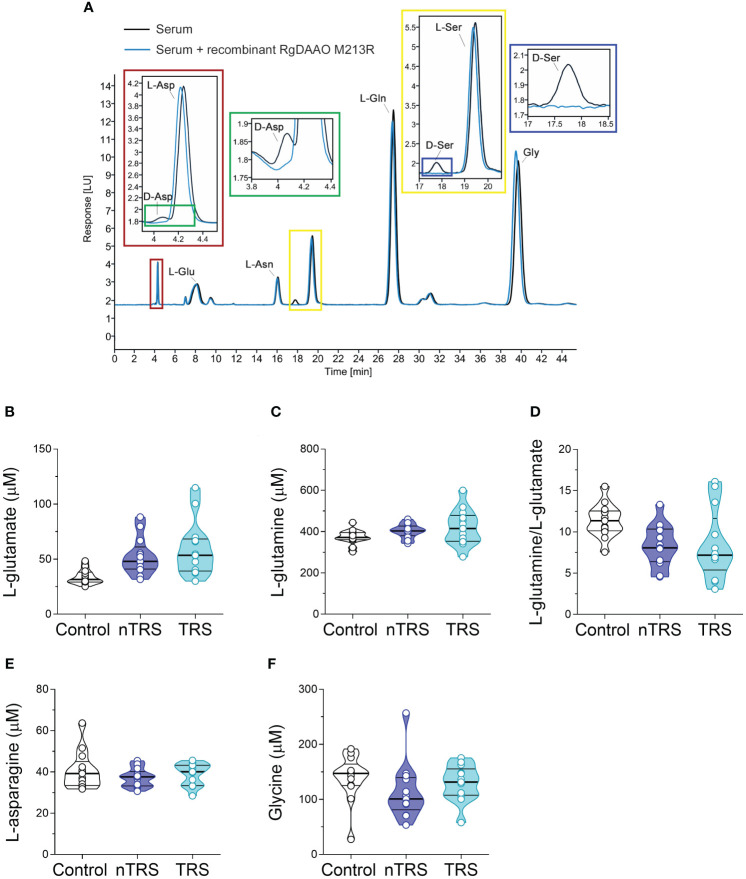
L-glutamate, L-glutamine, L-asparagine and glycine levels in the serum of schizophrenia patients and control subjects. **(A)** Overlaid HPLC chromatograms illustrating the specificity of D-aspartate (D-Asp) and D-serine (D-Ser) peaks obtained from blood serum samples. The identity of the peaks corresponding to D-aspartate and D-serine were verified by treating serum sample with RgDAAO M213R variant (a, inset, blu line). Analysis of **(B)** L-glutamate and **(C)** L-glutamine levels, **(D)** L-glutamine/L-glutamate ratio, **(E)** L-asparagine and **(F)** glycine levels in the serum of non-treatment-resistant schizophrenia (nTRS, *n* = 13) and treatment-resistant schizophrenia (TRS, *n* = 13) compared to control subjects (*n* = 13). The amino acid content was expressed as μM. In each sample, free amino acids were detected in a single run. Dots represent the single subjects’ values, while bars illustrate the median with interquartile range.

Serum samples were mixed in a 1:10 dilution with HPLC-grade methanol (900 µL) and centrifuged at 13,000 x g for 10 min. Supernatants were dried at 45°C and suspended in 0.2 M trichloroacetic acid (TCA). Samples were then neutralized with 0.2 M NaOH and subjected to pre-column derivatization with o-phthaldialdehyde/N-acetyl-L-cysteine in 50% methanol. To resolve diastereoisomer derivatives, two types of columns were used: a ZORBAX Eclipse Plus C8 5-μm reversed-phase column (Agilent, 4.6x250 mm) and a Symmetry C8 5 μm reversed-phase column (Waters, 4.6x250mm); the separation was performed under isocratic conditions (0.1 M sodium acetate buffer, pH 6.2, 1% tetrahydrofuran, 1 mL/min flow rate). A washing step in 0.1 M sodium acetate buffer, 3% tetrahydrofuran and 47% acetonitrile, was performed after every single run. Identification and quantification of D-Asp, L-aspartate (L-Asp), L-glutamate (L-Glu), L-asparagine (L-Asn), D-Ser, L-serine (L-Ser), L-glutamine (L-Gln) and glycine (Gly) were based on retention times and peak areas and then compared with those associated with external standards (**Figure 1A**). Peak’s identity was confirmed by the selective degradation of the D-enantiomers by RgDAAO M213R variant (**Figure 1A**) (88). Ten μg of the enzyme was added to the samples, incubated at 30°C for at least 3 h, and subsequently derivatized. Amino acids concentration in the serum were expressed as µM. D-amino acid/total amino acid ratio was expressed as a percentage (%). Quantification of enantiomers was based on peak areas using calibration curves for each enantiomer.

### 2.4 Statistical analysis

Data for clinical characteristics are reported as medians with the respective interquartile range (first-third quartile). The hypothesis of normality was assessed with the Shapiro-Wilk test. For variables not normally distributed, statistical analyses were performed using their log-transformed values. To identify potential confounders, we compared the demographic characteristics between patients and controls using two-sample (two-tailed) *t*-tests for age and Chi-square tests with Yates’ correction for sex. ANCOVA models (controlling for statistically different covariates between groups) were adopted to assess significant differences in amino acid concentrations between cases and controls. The *p*-values from ANCOVA models were corrected for multiple testing, following Bonferroni’s method. ANCOVAs were followed by Tukey *post-hoc* comparisons. To estimate the effect magnitude of significant outcomes, we computed the partial eta square (η²p), which provides a quantifiable measure of the proportion of variance in the dependent variable that is associated with an independent variable, while controlling for other variables. For Chi-square and *t*-test statistics, the Cohen’s w and d were computed as effect sizes, respectively. All statistical analyses were conducted with RStudio R version 4.1.2.

## 3 Results

### 3.1 L-glutamate serum levels show a trend toward an increase in schizophrenia patients

We recruited SCZ patients (*n* = 26) and non-psychiatric control subjects (*n* = 13) in A.O.U. “Federico II” Hospital (**Figure 1** **;** **Table 1**) to measure by HPLC the serum levels of L-Glu and Gly, which in the brain represent, respectively, the main excitatory amino acid and a major NMDAR co-agonist (together with D-Ser), as well as L-Gln and L-Asn, which participate to L-Glu and L-Asp biosynthesis, respectively. Specifically, SCZ patients were subdivided into nTRS and TRS groups (*n* = 13/condition) based on the assessment of symptoms persistence after at least two different conventional antipsychotic regimens (see Materials and Methods). No statistically significant differences were found in sex [χ^2^ (2, *N* = 39) = 5.0556, *p* = 0.0798, Cohen’s w = 0.36], while significant age-dependent variations were observed [median (min; max) of years: Ctrl = 28 (21; 40), nTRS = 47 (22; 60), TRS = 34 (25; 57), *F*(2, 36) = 10.688, *p* = 0.0002, η²p = 0.37, one-way ANOVA; **Table 1**] among groups. Based on this, to assess the changes in amino acid levels, we used an ANCOVA model considering the effect of age as a confounding factor.

We found significant differences in L-Glu serum content among groups [*F*(2, 35) = 5.552, *p* = 0.0082, η²p = 0.24], evidencing an increase of this amino acid in both nTRS and TRS patients, compared to controls (**Figures 1A, B** **;** **Supplementary Table 1**). However, such L-Glu serum variation did not survive after correction with the Bonferroni multiple comparisons method (**Supplementary Table 1**). Similarly, no alterations among groups were found for L-Gln levels, L-Gln/L-Glu ratio, L-Asn and Gly levels (**Figures 1A, C–F** **;** **Supplementary Table 1**). Overall, our analysis revealed no significant changes in L-Glu, L-Gln, L-Asn and Gly serum levels, as well as L-Gln/L-Glu serum ratio, in both nTRS and TRS patients, compared to their non-psychiatric controls.

### 3.2 Reduced D-serine and D-aspartate levels in the serum of schizophrenia patients

**Figure 2:**
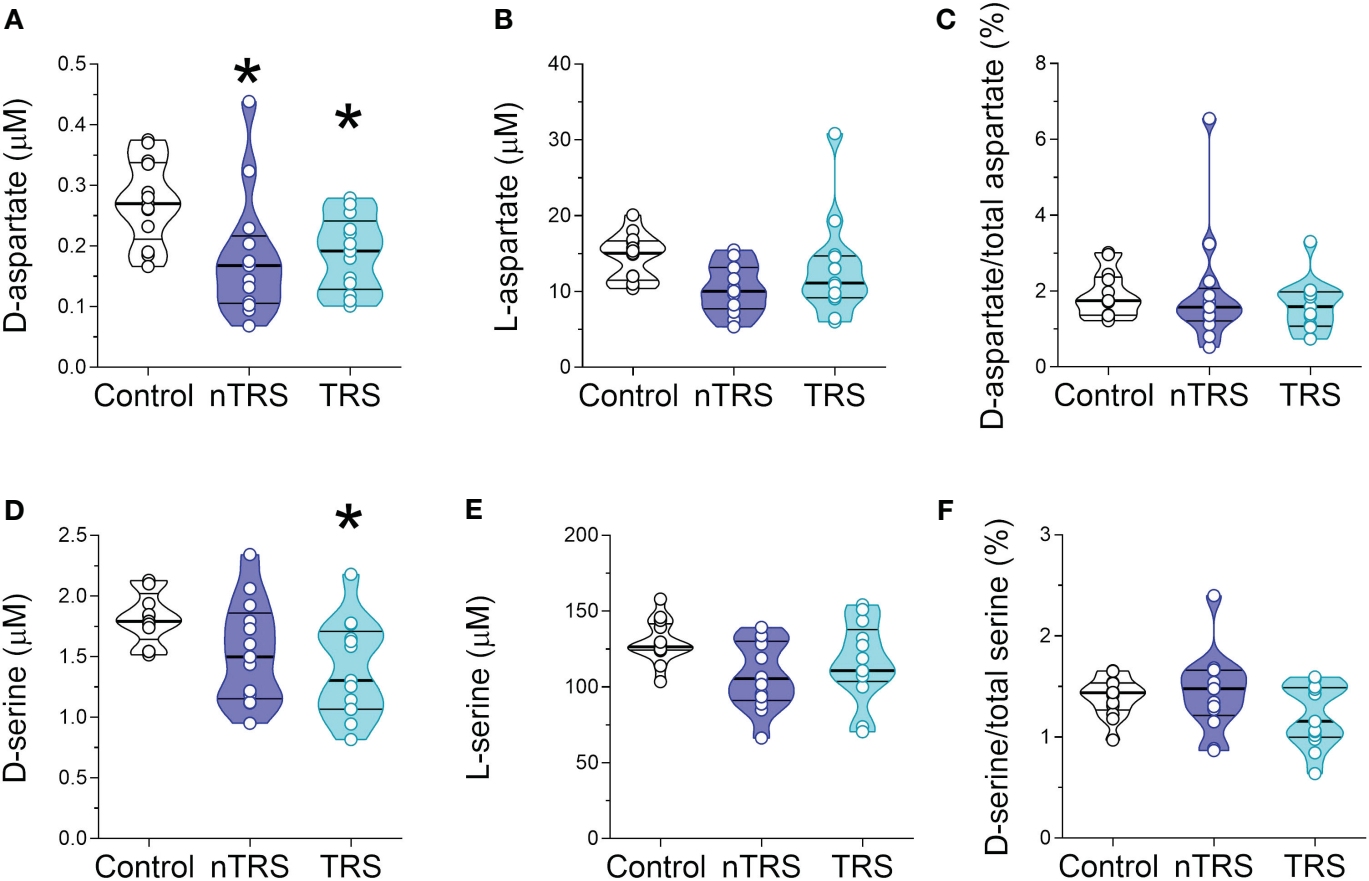
D-aspartate, L-aspartate, D-serine and L-serine levels in the serum of schizophrenia patients and control subjects. Analysis of **(A)** D-aspartate and **(B)** L-aspartate levels, **(C)** D-aspartate/total aspartate ratio, **(D)** D-serine and **(E)** L-serine levels, and **(F)** D-serine/total serine ratio in the serum of non-treatment-resistant schizophrenia (nTRS, *n* = 13) and treatment-resistant schizophrenia (TRS, *n* = 13) compared to control subjects (*n* = 13). The amino acid content was expressed as μM, while the ratios were expressed as percentages (%). In each sample, free amino acids were detected in a single run. Dots represent the single subjects’ values, while bars illustrate the median with interquartile range. *p <0.05, compared to the control group (Tukey *post-hoc* comparisons).

After we measured the levels of D-Ser, D-Asp and their respective precursors, L-Ser and L-Asp, the latter being also one of the major NMDAR agonists in the CNS. Statistical analysis revealed significant alteration in D-Asp levels in nTRS and TRS patients, compared to control individuals [*F*(2, 35) = 8.397, *p* = 0.001, η²p = 0.324], which survived also after correction with Bonferroni multiple comparisons (**Figures 2A** **;** **Supplementary Table 1**). The following Tukey *post-hoc* test highlighted that both nTRS and TRS patients displayed significantly reduced D-Asp levels, compared to controls (Ctrl vs nTRS, *p* = 0.0104; Ctrl vs TRS, *p* = 0.0177), while no significant alteration was observed between nTRS and TRS groups (TRS vs nTRS, *p* = 0.976) (**Figure 2A** **;** **Supplementary Table 1**). Conversely, we found comparable levels of L-Asp [*F*(2, 35) = 2.794, *p* = 0.0749] and D-Asp/total Asp ratio [*F*(2, 35) = 0.476, *p* = 0.6255] among nTRS, TRS and control subjects (**Figures 2B, C** **;** **Supplementary Table 1**).

Statistical analysis also revealed significant differences in D-Ser serum levels among nTRS, TRS patients and control subjects [*F*(2, 35) = 6.322, *p* = 0.0045, η²p = 0.265; **Figure 2D** **;** **Supplementary Table 1**), which was confirmed after correction with Bonferroni multiple comparisons (**Supplementary Table 1**). The following Tukey *post-hoc* analysis evidenced a significant decrease of D-Ser in TRS but not in nTRS patients, compared to controls (Ctrl vs nTRS, *p* = 0.1078; Ctrl vs TRS, *p* = 0.0103; **Figure 2D** **;** **Supplementary Table 1**). However, no D-Ser changes were found between TRS and nTRS patients (TRS vs nTRS, *p* = 0.569; **Figure 2D** **;** **Supplementary Table 1**). Also in this case, the deregulation was confined to the D-enantiomer levels, as L-Ser levels did not significantly change among groups [*F*(2, 35) = 2.900, *p* = 0.0683; **Figure 2E** **;** **Supplementary Table 1**]. Despite the decrease being specific for the D-Ser, we found an unaltered D-Ser/total Ser ratio [*F*(2, 35) = 1.757, *p* = 0.1875; **Figure 2F** **;** **Supplementary Table 1**].

Altogether, our analyses showed selective reductions in D-Asp serum levels in both nTRS and TRS patients, and in D-Ser serum levels only in TRS group, compared to non-psychiatric control subjects. Conversely, no alterations were found in their respective L-enantiomers, L-Asp and L-Ser, among nTRS, TRS and control individuals.

### 3.3 Unaltered L-glutamate, L-glutamine, L-asparagine and glycine levels in the serum of ASD patients

**Figure 3:**
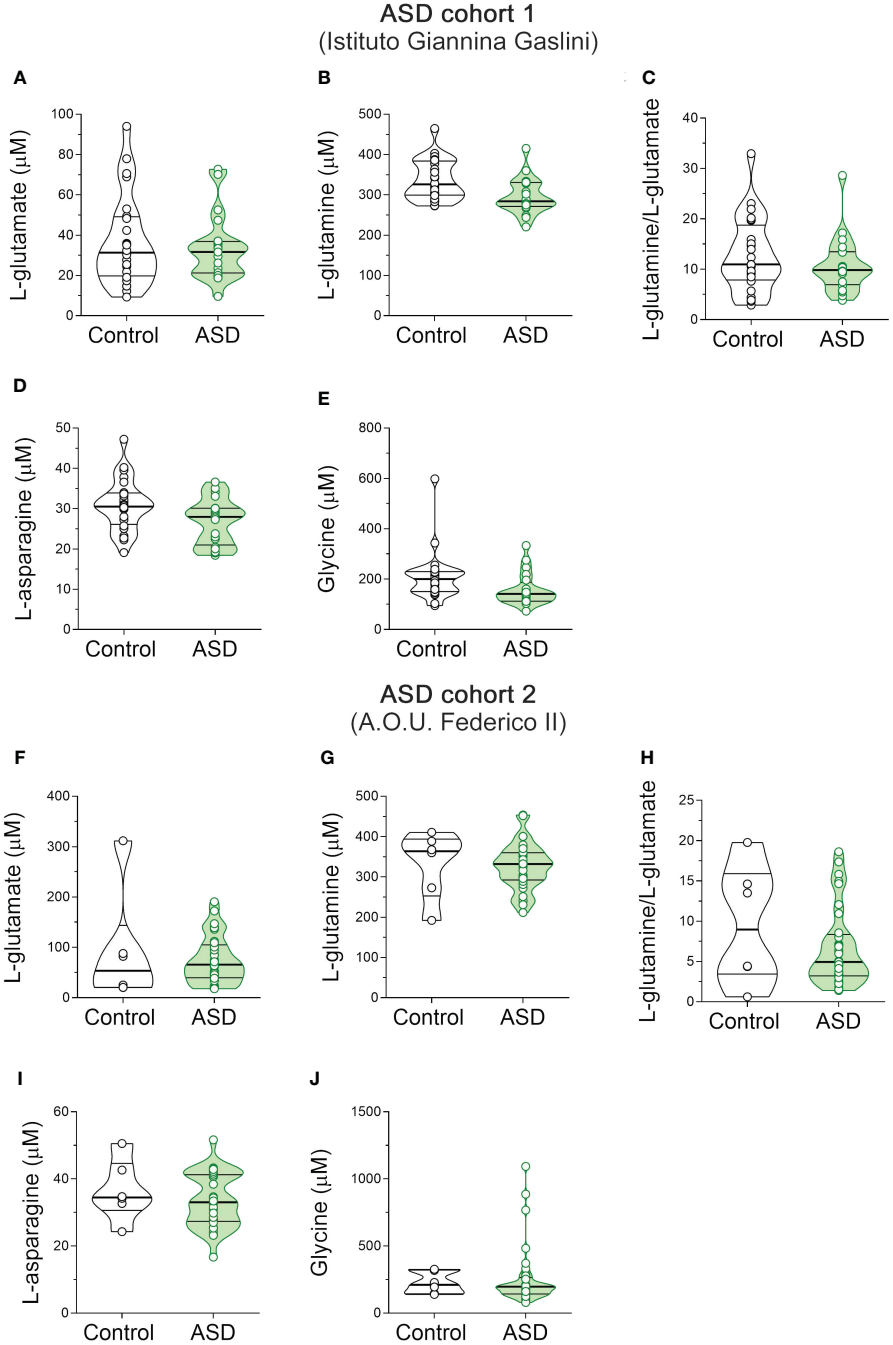
L-glutamate, L-glutamine, L-asparagine and glycine content in the serum of autism spectrum disorder patients. Analysis of **(A, F)** L-glutamate and **(B, G)** L-glutamine levels, **(C, H)** L-glutamine/L-glutamate ratio, **(D, I)** L-asparagine and **(E, J)** glycine levels in the serum of autism spectrum disorder (ASD) patients compared to control subjects enrolled in **(A–E)** Istituto Giannina Gaslini (Control, *n* = 24; ASD, *n* = 20) and **(F–J)** A.O.U. Federico II (Control, *n* = 6; ASD, *n* = 33) hospitals. The amino acid content was expressed as μM. In each sample, free amino acids were detected in a single run. Dots represent the single subjects’ values, while bars illustrate the median with interquartile range.

Then we measured the levels of the same neuroactive amino acids and their precursors in the serum of pediatric ASD patients and control subjects recruited in two different Italian Hospitals (Istituto Giannina Gaslini: ASD, *n* = 20, Ctrl, *n* = 24; A.O.U. “Federico II”: ASD, *n* = 33; Ctrl, *n* = 6). First, we analyzed the cohort of ASD patients and control subjects from Istituto Giannina Gaslini. Before proceeding with statistical comparisons, we assessed potential imbalance in clinical variables, such as sex and age, between ASD patients and control individuals. No statistically significant differences were found in sex (χ^2^ (1, *N* = 44) = 0.009, *p* = 0.926), while significant variations between groups were observed in age [median (min; max) of years: Ctrl = 9.8 (3.1; 24.8) vs ASD = 6.1 (3.5; 11.3), *t*(42) = -3.414, *p* = 0.001, Cohen’s d = -1.03; Student’s *t* test] (**Table 2**). Based on this, to assess amino acid variations between ASD and control subjects, we used ANCOVA model, including age as a confounder. Statistical analysis revealed no significant alterations in L-Glu [*F*(1, 41) = 0.769, *p* = 0.3855] and L-Gln [*F*(1, 41) = 2.127, *p* = 0.1524] levels, L-Gln/L-Glu ratio [*F*(1, 41) = 0.285, *p* = 0.5961], as well as L-Asn [*F*(1, 41) = 1.785; *p* = 0.1889] and Gly [*F*(1, 41) = 2.143; *p* = 0.1508] levels between the two diagnosis groups (**Figures 3A–E**, **Supplementary Table 2**).

Then, we analyzed the cohort of ASD patients and control subjects recruited from A.O.U. “Federico II” hospital. We observed significant differences in both sex (χ^2^ (1, *N* = 39) = 9.060; *p* = 0.003, Cohen’s w = 0.56) and age [median (min; max) of years: Ctrl = 13 (5; 17) vs ASD = 5.4 (0.4; 15.7); *t*(36) = -3.075, *p* = 0.004, Cohen’s d = -1.37; Student’s *t* test] (**Table 2**). For this reason, we evaluated the differences between groups by ANCOVA models, including both age and sex as confounders. HPLC analysis revealed unaltered levels of each of the analyzed amino acids [L-Glu: *F*(1, 34) = 3.307, *p* = 0.0778; L-Gln: *F*(1, 34) = 1.504, *p* = 0.2285; L-Gln/L-Glu ratio: *F*(1, 34) = 0.170, *p* = 0.6825; L-Asn *F*(1, 34) = 1.188, *p* = 0.2834; Gly: *F*(1, 34) = 0.044, *p* = 0.8346] (**Figures 3F–J**, **Supplementary Table 2**) in ASD patients, compared to control subjects. Collectively, our results show no significant alterations in L-Glu, L-Gln, L-Asn and Gly serum levels, as well as L-Gln/L-Glu serum ratio, in both cohorts of ASD patients analyzed, compared to their respective control individuals.

### 3.4 Unaltered D-serine, D-aspartate and their L-enantiomers levels in the serum of ASD patients

**Figure 4:**
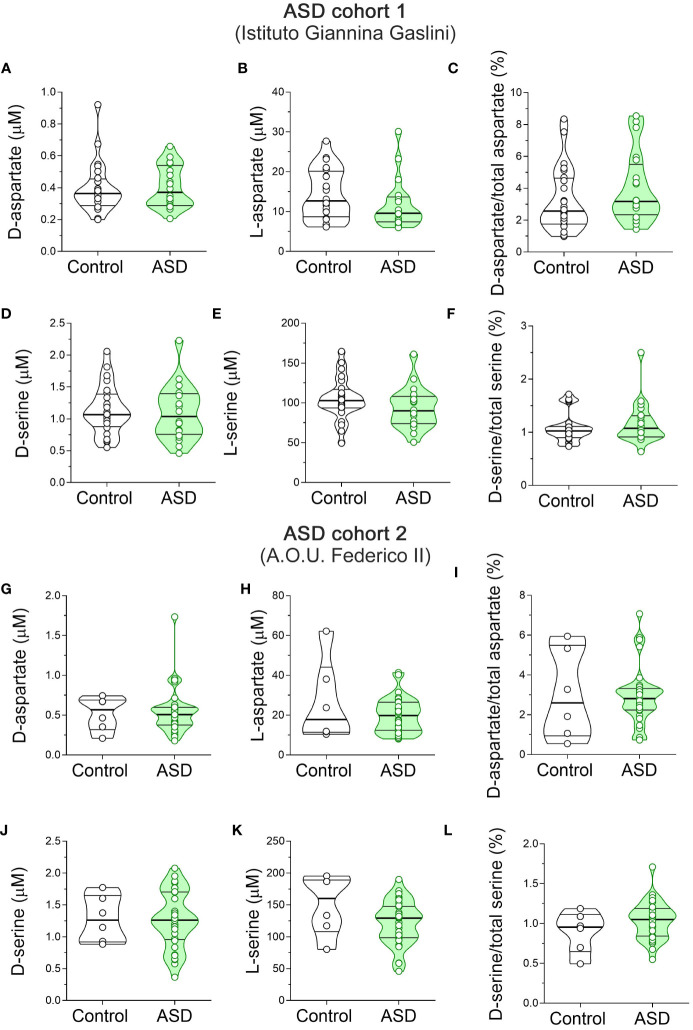
D-aspartate, L-aspartate, D-serine and L-serine levels in the serum of autism spectrum disorder patients and control subjects. Analysis of **(A, G)** D-aspartate and **(B, H)** L-aspartate levels, **(C, I)** D-aspartate/total aspartate ratio, **(D, J)** D-serine and **(E, K)** L-serine levels, and **(F, L)** D-serine/total serine ratio in the serum of autism spectrum disorder (ASD) patients compared to control subjects enrolled by **(A–F)** Istituto Giannina Gaslini (Control, *n* = 24; ASD, *n* = 20) and **(G–I)** A.O.U. Federico II (Control, *n* = 6; ASD, *n* = 33) hospitals. The amino acid content was expressed as μM, while the ratios were expressed as percentages (%). In each sample, free amino acids were detected in a single run. Dots represent the single subjects’ values, while bars illustrate the median with interquartile range. .

Finally, we measured the serum content of D-Ser, L-Ser, D-Asp and L-Asp. Statistical analysis in ASD patients and control subjects from Istituto Giannina Gaslini revealed no significant differences in D-Asp (*F*(1, 41) = 0.046, p = 0.8317), L-Asp (*F*(1, 41) = 1.443, *p* = 0.2366), and D-Asp/total Asp ratio (*F*(1, 41) = 0.561, *p* = 0.4583) between ASD patients and control subjects (**Figures 4A–C**, **Supplementary Table 2**). Likewise, we found comparable levels between diagnoses also for D-Ser (*F*(1, 41) = 0.235, *p* = 0.6301), L-Ser (*F*(1, 41) = 0.659, *p* = 0.4218) and D-Ser/total Ser ratio (*F*(1, 41) = 0.058, *p* = 0.8113) (**Figures 4D–F**, **Supplementary Table 2**). Finally, we analyzed ASD patients and control subjects from A.O.U. “Federico II”. We found unaltered D-Asp levels between diagnoses (*F*(1, 41) = 0.036, *p* = 0.8507; **Figure 4G**, **Supplementary Table 2**). Conversely, we showed a slight but significant L-Asp levels increase in ASD patients, compared to controls (*F*(1, 34) = 4.449, *p* = 0.0424; η²p = 0.12; **Figure 4H**, **Supplementary Table 2**), which did not survive after correction with Bonferroni multiple comparisons method (**Supplementary Table 2**). In line with unaltered D-Asp and L-Asp levels, D-Asp/total Asp ratio did not significantly change between diagnosis groups (*F*(1, 34) = 0.0001 *p* = 0.9954; **Figure 4I**, **Supplementary Table 2**). Again, ANCOVA analysis revealed comparable D-Ser and L-Ser levels [*F*(1, 34) = 0.027, *p* = 0.8704], L-Ser [*F*(1, 34) = 3.132, *p* = 0.0858], and D-Ser/total Ser ratio [*F*(1, 34) = 1.347; *p* = 0.2539] (**Figures 4J–L**, **Supplementary Table 2**) between ASD patients and control individuals. Our overall HPLC analysis revealed no significant changes in D-Asp, L-Asp, D-Ser and L-Ser serum levels, as well as D-Asp/total Asp and D-Ser/total Ser serum ratios, in both cohorts of ASD patients analyzed, compared to their respective control individuals.

## 4 Discussion

In the present work, we aimed to measure the serum levels of the major neuroactive amino acids acting in glutamatergic system in patients with ASD and SCZ, compared to healthy controls. Overall, in ASD patients, recruited in two different Medical Centers, we failed to reveal significant variations in serum levels of all the amino acids detected. Previous findings have shown contrasting results on this subject (89). Indeed, some studies are in line with our data as they revealed unaltered Gln, Gly, Asp, L-Ser and D-Ser levels in the blood of ASD patients, compared to control subjects (90, 91). Conversely, increased Glu, Asp, Asn (90–100) or decreased Gln and Asn (90, 92–95, 98, 100, 101) levels have been reported in other investigations. Methodological issues and the sample size of the cohorts of patients included in the different studies may explain these discrepancies (89). Furthermore, divergent clinical phenotypes may also cause inconsistency, as ASD features are highly heterogeneous in terms of clinical presentation, genetic variability, and comorbidity (102).

Unlike ASD, HPLC analysis revealed variations in both D-Asp and D-Ser serum levels in SCZ patients, compared to non-psychiatric controls. In particular, we detected significantly lower D-Ser serum levels in TRS, and a trend toward reduction in the nTRS group, compared to controls. D-Ser levels were comparable between TRS and nTRS patients. No statistically significant changes in L-Ser levels occurred between clinical conditions. These results are in agreement with other studies revealing decreased peripheral blood levels of D-Ser in SCZ patients (43, 44, 48–55). Noteworthy, this is the first study providing peripheral blood levels of D-Asp and its ratio with total Asp in SCZ patients. Interestingly, we detected a significant downregulation in D-Asp serum levels in both TRS and nTRS patients relative to controls. In contrast, the serum concentration of the putative D-Asp precursor, L-Asp, and the D-Asp/total Asp ratio did not change among clinical conditions.

The decreased D-Ser and D-Asp serum levels reported in the current investigation may reflect previous evidence of altered levels of both D-amino acids in the brain and CSF of SCZ patients, compared to non-psychiatric subjects. Indeed, lower D-Ser levels and/or D-Ser/total Ser ratio were found in two independent CSF cohorts of SCZ patients (46, 47). Likewise, a significant downregulation of D-Asp levels and D-Asp/total Asp ratio was found in two independent cohorts of *post-mortem* PFC samples from SCZ patients, associated with either increased *DDO* gene expression (103) or enzymatic DDO activity (76). In line with a possible D-Asp metabolism dysfunction in neurodevelopmental processes, a recent study reported the first case of *DDO* gene duplication in a young patient with clinical manifestations resembling both ASD and SCZ symptoms (78).

Several studies support the hypothesis that NMDAR hypofunction in the SCZ brain accounts for cognitive and attentional deficits commonly reported in patients (104). Based on this assumption, abnormally lower cerebral D-Ser and D-Asp levels in the developing brain might produce NMDAR abnormalities and, in turn, contribute to SCZ pathophysiology (6, 26, 105). However, despite the existence of a correlation between blood and CSF amino acid levels, including glycine and serine (106), and the ability of D-Ser and D-Asp to cross the blood-brain barrier (107–109), it is yet unknown whether the circulating amounts of D-Ser and D-Asp may reflect the synaptic concentrations of these endogenous NMDAR signaling molecules in the SCZ brain.

We argue that lower serum levels of D-Ser and D-Asp, but not of their corresponding L-enantiomers, in SCZ patients may depend on the complex dynamics of D-amino acid metabolism in both brain and peripheral organs (38), thus possibly implying dysfunction of the catabolic enzymes, DDO and DAAO, the DAAO modulator pLG72, and the biosynthetic enzyme SR, the latter being also involved in D-Asp production, at least in the mouse forebrain regions (33, 34).

Besides canonical endogenous sources, recent work revealed that an important contribution to systemic D-amino acid level variations originates from the intestine (37), which regulates the absorption of diet- and microbial-derived exogenous amino acids. In this organ, the abundance of bacterial D-amino acids exerts a key role in modulating mammalian immune responses and symbiosis with bacteria (110). Therefore, any event producing dysbiosis, such as inflammation or pharmacological treatments, may alter peripheral and central D-Ser and D-Asp homeostasis as both D-amino acids cross the blood-brain barrier (108, 109). Since an increased prevalence of neuroinflammation and autoimmune disorders is well documented in SCZ (111), we hypothesize that these conditions may contribute to the altered D-Ser and D-Asp serum levels reported in patients.

Another important issue is whether D-Ser and D-Asp serum levels are linked to antipsychotic treatments. A longitudinal analysis of D-Ser plasma levels in TRS patients revealed a comparable reduction of this D-amino acid either before or after clozapine treatment, relative to baseline, despite the decrease being statistically significant only before clozapine administration (51). This evidence would suggest that clozapine administration does not contribute to the specific D-Ser serum level reduction observed in our TRS patients, as they were all under treatment with this antipsychotic. Another study evidenced increased serum levels of different amino acids, among which Asp, Ser, Glu and Gly, in SCZ patients treated with clozapine, relative to those treated with conventional antipsychotics (112). However, a comparison with our study is challenging since the work of Melkersson et al. lacks a reference non-psychiatric control group to assess basal amino acid concentrations and does not discriminate between D- and L-enantiomers. Finally, although restricted to the CNS of preclinical models, our and other studies revealed an effect of clozapine and olanzapine on the brain availability of D-Ser and D-Asp, respectively. Indeed, it has been reported that clozapine modulates extracellular D-Ser release in the medial PFC of freely moving rats (113), while olanzapine affects D-Asp metabolism in the mouse brain by inhibiting enzymatic DDO activity (109).

A recent line of research is bringing attention to the influence of antipsychotic medications on the composition of gut microbiota (114), thus potentially linking their use to changes in systemic D-amino acids availability. In particular, a recent study reported differentially abundant bacterial species in the gut microbiota of individuals with SCZ responding or resistant to antipsychotic treatment compared to healthy controls suggesting a potential role of clozapine in TRS patients (115). Future studies on larger cohorts of nTRS and TRS patients extended to first-episode patients and at-risk mental state individuals, will be needed to help clarify the still unclear role of antipsychotics on D-amino acid metabolism. Additionally, blood D-Ser levels are strongly correlated with glomerular filtration rate, emerging as a potential biomarker of kidney functionality (116, 117). As SCZ patients are more likely to have comorbid chronic kidney diseases (118), the potential bias of kidney dysfunction should be taken into account when considering peripheral D-Ser changes in SCZ patients.

Here, we also found a remarkable trend towards L-Glu upregulation in the serum of both nTRS and TRS patients, compared to controls. In line with present data, previous findings revealed that peripheral blood variations of this excitatory amino acid may reflect antipsychotic therapy (112, 119–122).

The major limitation of this work is the relatively small sample size of the ASD and SCZ patient cohorts and their relative controls. In the ASD study, such constraint is partially attenuated by recruiting two independent cohorts of patients and respective control subjects, whose HPLC serum detections revealed comparable results for all the amino acids analyzed.

To our knowledge, the present work represents the first serum detection of D-Asp in ASD and SCZ patients, as prior studies did not differentiate the relative contribution of D- and L-stereoisomers. Another strength point of our study is the simultaneous analysis of all the main D- and L-excitatory amino acids implicated in NMDAR transmission and their precursors. However, as L-Glu is also the precursor of the principal inhibitory neurotransmitter of the CNS, the gamma amino-butyric acid (GABA), future studies are required to evaluate the contribution of inhibitory neurotransmission in developmental psychiatric disorders and understand whether D-Ser and D-Asp serum levels may change in SCZ patients as compensatory events for potential inhibitory system dysfunctions.

In conclusion, further studies on new and more numerous cohorts of patients are needed to confirm the unaltered amino acid levels found in the serum of ASD patients, compared to controls. Conversely, the detection of lower D-Ser and D-Asp serum levels in SCZ patients encourages future research aimed at evaluating the potential role of these atypical amino acids as *in vivo* biochemical markers for alteration of the glutamatergic system in SCZ and association of putative differential changes of D-Ser and D-Asp levels with different classes of antipsychotics and response to treatment.

## Data availability statement

The raw data supporting the conclusions of this article will be made available by the authors, without undue reservation.

## Ethics statement

The studies involving humans were approved by Ethics Committee of the University “Federico II” of Naples (protocol number: 195/19); Ethics Committee of the University Federico II of Naples (220/18); Ethics Committee of the Liguria Region (N. CET - Liguria: 437/2023 - DB id 13411). The studies were conducted in accordance with the local legislation and institutional requirements. Written informed consent for participation in this study was provided by the participants’ legal guardians/next of kin.

## Author contributions

MG: Investigation, Writing – original draft. GD: Investigation, Writing – original draft. ZM: Investigation, Writing – original draft. TN: Investigation, Writing – original draft. ED: Resources, Writing – review & editing. CB: Resources, Writing – review & editing. SB: Resources, Writing – review & editing. MR: Resources, Writing – review & editing. LP: Writing – review & editing. CB: Resources, Writing – review & editing. FI: Resources, Writing – review & editing. FS: Conceptualization, Writing – review & editing. LP: Conceptualization, Funding acquisition, Supervision, Validation, Writing – review & editing. FE: Conceptualization, Funding acquisition, Supervision, Writing – review & editing. AD: Conceptualization, Funding acquisition, Resources, Supervision, Writing – review & editing. AU: Conceptualization, Funding acquisition, Project administration, Supervision, Writing – review & editing.
