# Supplementary material for: Decreased free D-aspartate levels in the blood serum of patients with schizophrenia
Source: Front Psychiatry. 2024 Jul 10;15:1408175. doi: 10.3389/fpsyt.2024.1408175 (PMC11266155; doi:10.3389/fpsyt.2024.1408175)
Supplement: Supplementary file 1 [file Table_1.docx]

Supplementary Material

# Supplementary Tables

| **Supplementary table 1**. Comparison of serum amino acid levels among nTRS, TRS and control patients. | | | | | | | | | |
| --- | --- | --- | --- | --- | --- | --- | --- | --- | --- |
| **Amino acid (µM)** | **Control (n=13)** | **nTRS (n=13)** | **TRS (n=13)** | **ANCOVA** | | **Bonferroni** | **Tukey *post-hoc* (*p*-value)** | | |
|  | **Median [min; max]** | | | **F _(2,35)_** | ***p*-value** | ***Adjusted p*-value** | **Control vs nTRS** | **Control *vs* TRS** | **TRS *vs* nTRS** |
| D-aspartate | 0.3 [0.2;0.4] | 0.2 [0.1;0.4] | 0.2 [0.1;0.3] | 8,397 | 0.001 | **0.0110** | 0.010***** | 0.018***** | 0.976 |
| L-aspartate | 15.1 [10.4;20.1] | 10 [5.3;15.5] | 11.1 [6;30.8] | 2,794 | 0.0749 | 0.8239 |  |  |  |
| D-aspartate/total aspartate (%) | 1.8 [1.2;3] | 1.6 [0.5;6.5] | 1.6 [0.7;3.3] | 0.476 | 0.6255 | 1.0000 |  |  |  |
| L-glutamate | 31.6 [25.2;48.4] | 48 [31.7;88.2] | 53.4 [30;114.9] | 5,522 | 0.0082 | 0.0902 |  |  |  |
| L-glutamine | 371.7 [302.2;444.4] | 403.1 [343.4;460.2] | 415 [277.6;600.3] | 1,685 | 0.2001 | 1.0000 |  |  |  |
| L-glutamine/L-glutamate | 11.3 [7.6;15.5] | 8.1 [4.5;13.3] | 7.2 [3.1;16.1] | 2,409 | 0.1047 | 1.0000 |  |  |  |
| L-asparagine | 39.2 [31.8;63.6] | 37.6 [30.6;45.4] | 40.1 [28.4;45.6] | 0.315 | 0.7316 | 1.0000 |  |  |  |
| D-serine | 1.8 [1.5;2.1] | 1.5 [1;2.3] | 1.3 [0.8;2.2] | 6,322 | 0.0045 | **0.0495** | 0.108 | 0.010***** | 0.569 |
| L-serine | 126.4 [103.5;157.9] | 105.4 [66.3;139.3] | 110.8 [70.5;154.3] | 2,900 | 0.0683 | 0.7513 |  |  |  |
| D-serine/total serine (%) | 1.4 [1;1.7] | 1.5 [0.9;2.4] | 1.2 [0.6;1.6] | 1,757 | 0.1875 | 1.0000 |  |  |  |
| Glycine | 147.2 [27.3;191.8] | 100.6 [53.4;257.1] | 131.5 [57.9;175.6] | 1,643 | 0.2079 | 1.0000 |  |  |  |
| Statistical analyses were performed by ANCOVA considering the effect of age, followed by Tukey *post-hoc* multiple comparisons when required (*****p<0.05). Adjusted p-values correspond to *p*-values corrected for multiple testing following the Bonferroni method (significant *p*-values are shown in bold). Abbreviations: nTRS: non-treatment-resistant schizophrenia, TRS: treatment-resistant schizophrenia. | | | | | | | | | |

| **Supplementary table 2**. Comparison of serum amino acid levels between ASD patients and control subjects enrolled in two different Italian hospitals. | | | | | | | | | |  |
| --- | --- | --- | --- | --- | --- | --- | --- | --- | --- | --- |
|  | **Istituto Giannina Gaslini** | | | | | **A.O.U. "Federico II"** | | | | |
| **Amino acid (µM)** | **Control (n=24)** | **ASD (n=20)** | **ANCOVA^a^** | | **Bonferroni** | **Control (n=6)** | **ASD (n=33)** | **ANCOVA^b^** | | **Bonferroni** |
|  | ***Median [min; max]*** | | ***F_(1,41)_*** | ***p-value*** | ***p-value adjusted^#^*** | ***Median [min-max]*** | | ***F_(1,34)_*** | ***p-value*** | ***p-value adjusted^#^*** |
| D-aspartate | 0.4 [0.2;0.9] | 0.4 [0.2;0.7] | 0.046 | 0.8317 | 1.0000 | 0.6 [0.2;0.7] | 0.5 [0.2;1.7] | 0.036 | 0.8507 | 1.0000 |
| L-aspartate | 12.7 [6.2;27.7] | 9.6 [6;30.1] | 1,443 | 0.2366 | 1.0000 | 17.9 [10.5;62.2] | 19.9 [8.1;41.4] | 4,449 | 0.0424 | 0.4664 |
| D-aspartate/total aspartate (%) | 2.6 [1;8.3] | 3.2 [1.4;8.5] | 0.561 | 0.4583 | 1.0000 | 2.6 [0.5;5.9] | 2.8 [0.7;7.1] | 0.000 | 0.9954 | 1.0000 |
| L-glutamate | 31.3 [9.3;94] | 31.7 [9.6;72.8] | 0.769 | 0.3855 | 1.0000 | 53.8 [20.2;311.7] | 65.9 [17.9;190.4] | 3,307 | 0.0778 | 0.8558 |
| L-glutamine | 326.0 [272.7;464.6] | 284 [220.6;415.3] | 2,127 | 0.1524 | 1.0000 | 363.8 [192.1;410.5] | 332 [211.6;453.6] | 1,504 | 0.2285 | 1.0000 |
| L-glutamine/L-glutamate | 11.0 [2.9;33] | 9.9 [3.9;28.6] | 0.285 | 0.5961 | 1.0000 | 9.0 [0.6;19.8] | 4.9 [1.4;18.6] | 0.170 | 0.6825 | 1.0000 |
| L-asparagine | 30.5 [19.1;47.2] | 27.9 [18.4;36.6] | 1,785 | 0.1889 | 1.0000 | 34.5 [24.3;50.6] | 33.0 [16.7;51.7] | 1,188 | 0.2834 | 1.0000 |
| D-serine | 1.1 [0.5;2.1] | 1.0 [0.5;2.2] | 0.235 | 0.6301 | 1.0000 | 1.3 [0.9;1.8] | 1.3 [0.4;2.1] | 0.027 | 0.8704 | 1.0000 |
| L-serine | 102.9 [49.4;164.6] | 90.0 [50.5;161.1] | 0.659 | 0.4218 | 1.0000 | 160.1 [80.4;195.4] | 129.2 [45.9;189.8] | 3,132 | 0.0858 | 0.9438 |
| D-serine/total serine (%) | 1.0 [0.7;1.7] | 1.1 [0.6;2.5] | 0.058 | 0.8113 | 1.0000 | 1.0 [0.5;1.2] | 1.1 [0.5;1.7] | 1,347 | 0.2539 | 1.0000 |
| Glycine | 200.3 [94.9;598.6] | 141.7 [73.1;333.6] | 2,143 | 0.1508 | 1.0000 | 211.4 [139.7;327] | 197.8 [79.8;1093.6] | 0.044 | 0.8346 | 1.0000 |
| Values are expressed as median [minimun; maximum]. Number of subjects (n) is indicated. Statistical analyses were performed by ANCOVA considering the effect of ^a^age or ^b^age and sex. ^#^Adjusted p-values correspond to *p*-values corrected for multiple testing following the Bonferroni method. Abbreviations: Autism spectrum disorder (ASD). | | | | | | | | | | |
